# Identification of WxL and S-Layer Proteins from *Lactobacillus brevis* with the Ability to Bind Cellulose and Xylan

**DOI:** 10.3390/ijms23084136

**Published:** 2022-04-08

**Authors:** Zhenzhen Hao, Wenjing Zhang, Xiaolu Wang, Yuan Wang, Xing Qin, Huiying Luo, Huoqing Huang, Xiaoyun Su

**Affiliations:** State Key Laboratory of Animal Nutrition, Institute of Animal Sciences, Chinese Academy of Agricultural Sciences, Beijing 100193, China; zhenzhenhao2012@163.com (Z.H.); 15927146115@163.com (W.Z.); xiaolu4444@126.com (X.W.); wangyuan08@caas.cn (Y.W.); qinxing@caas.cn (X.Q.); luohuiying@caas.cn (H.L.)

**Keywords:** gut bacteria, *Lactobacillus brevis*, WxL protein, S-layer protein, xylanase, cell adhesion

## Abstract

Xylanase releases xylo-oligosaccharides from dietary xylan, which stimulate the growth of the gut bacteria lactobacilli. Many lactobacilli adhere to dietary fibers, which may facilitate the assimilation of xylo-oligosaccharides and help them gain competence in the gut, but the underlying mechanisms remain elusive. Herein we report, from the highly abundant transcripts of *Lactobacillus brevis* cultured in wheat arabinoxylan supplemented with a xylanase, the identification of genes encoding four putative cell-surface WxL proteins (Lb630, Lb631, Lb632, and Lb635) and one S-layer protein (Lb1325) with either cellulose- or xylan-binding ability. The repetitively occurring WxL proteins were encoded by a gene cluster, among which Lb630 was chosen for further mutational studies. The analysis revealed three aromatic residues (F30, W61, and W156) that might be involved in the interaction of the protein with cellulose. A homology search in the genome of *Enterococcus faecium* identified three WxL proteins with conserved counterparts of these three aromatic residues, and they were also found to be able to bind cellulose and xylan. The findings suggested a role of the cell-surface WxL and S-layer proteins in assisting the cellular adhesion of *L. brevis* to plant cell wall polysaccharides.

## 1. Introduction

For many microbes living in the guts of humans and animals, the intestinal niche, which normally houses up to 100 billion microorganisms, is a highly competitive environment. The competition between two gut bacteria may be manifested by one bacterium’s excretion of toxic molecules, such as bacteriocin, that are lethal to the other [1,2]. However, competition can also be demonstrated by distinctive efficiencies in the utilization of specific nutrients in the culture medium, leading to the outgrowth of one bacterium over the others in the co-culture [3].

Plant cell wall polysaccharides (PCWP), or referred to as dietary fibers, are examples of nutrients [4] in the gut niche that favor the growth of specific gut bacteria [5,6,7]. Xylanase is an enzyme that degrades xylan (the second most abundant PCWP) and releases xylo-oligosaccharides supporting growth of certain gut bacteria. When used to supplement wheat-based animal feed, xylanase has been repetitively reported to be beneficial for livestock and aquatic animals, such as broiler chickens, gilt pigs, and croakers [8,9,10]. This beneficence stems from the enhanced breakage of plant cell walls, which facilitates the release of the starch and protein embedded in the wheat cells. Moreover, the addition of xylanase in feed is observed to alter the gut microbiota, in which the abundance of lactobacilli has been frequently found to be elevated [11,12,13]. The selective enrichment of lactobacilli by xylanase is of significant importance to animals, since these bacteria are normally regarded as probiotics. Intriguingly, some lactobacilli strains are among the few major causative strains responsible for alleviating the symptoms of type 2 diabetes in humans [5], suggesting the usefulness of xylanase in areas beyond the livestock industry. The versatile lactobacilli [14] are organisms that degrade and utilize xylo-oligosaccharides, which are generated by the xylanase-catalyzed depolymerization of the polysaccharide xylan. Interestingly, some strains of these gut bacteria can adhere to dietary fibers [15,16] reminiscent of the gut bacteria such as *Fibrobacter succinogenes* and *Ruminococcus albus*, which gain in competence by sticking to dietary fibers and avoiding oligosaccharide nutrient flows [17].

The mechanisms underlying the binding of lactobacilli to PCWP are poorly understood. Some gut bacteria feature cell-surface WxL proteins, which are non-covalently anchored to cell-wall peptidoglycans [18,19]. In *Enterococcus faecium*, three WxL proteins (DufA, LwpA, and SwpA) could bind type I collagen and fibronectin [19]. Although the binding mechanisms remain to be elucidated, we noted that, unlike DufA and LwpA, SwpA appeared to be composed of a single WxL domain. This suggested that the observed binding to the two human extracellular matrix proteins could take place through this WxL domain [19]. In addition, the bacterial surface (S) layer proteins (i.e., S-layer proteins, such as the SLH domain-containing proteins) that mediate the attachment of the bacterium to the extracellular matrix components and the gut epithelial cells [20,21]. Herein, we investigate whether four single WxL domain-containing proteins and an S-layer protein, found to be encoded in highly transcribed genes from *L. brevis* cultured in wheat arabinoxylan supplemented with xylanase, can bind to two polysaccharide substrates, i.e., cellulose and xylan (the two main components of PCWP), and discuss their role in facilitating the cell adhesion of *L. brevis* to plant cell wall polysaccharides.

## 2. Results

### 2.1. L. brevis Binds Crystalline Cellulose

**Figure 1:**
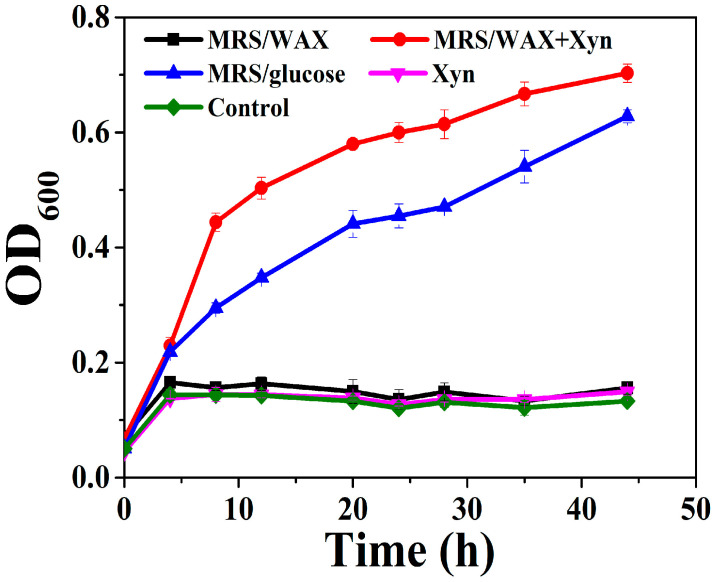
Growth of *L. brevis* in MRS supplemented with different carbon sources. WAX: wheat arabinoxylan; WAX+Xyn: wheat arabinoxylan plus xylanase; Control: no carbon source.

**Figure 2:**
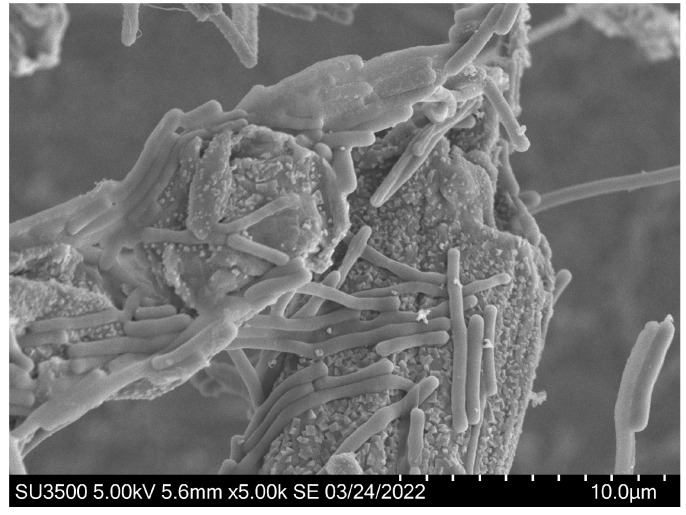
Scanning electron microscopy (SEM) analysis indicated that *L. brevis* cells were directly attached to the crystalline cellulose. The bars indicate 10.0 μm.

A *L. brevis* strain was isolated from the intestinal content of a broiler chicken. The *L. brevis* did not grow significantly in the MRS media supplemented with no saccharide or with only WAX (MRS/WAX). However, it grew rapidly in the MRS supplemented with glucose (abbreviated as MRS/glucose) and even faster in the MRS supplemented with WAX and xylanase (MRS/WAX+Xyn) (Figure 1). The genomic sequencing of this strain indicated that three xylosidases, two arabinofuranosidases, one α-glucuronidase, and one esterase, but no xylanase, are intracellular and putatively involved in xylooligosaccharide degradation (to be published elsewhere). The presence of these enzymes underpinned the growth of the strain on the MRS/WAX+Xyn, which produced xylooligosaccharides as substrates of these enzymes. Therefore, the *L. brevis* was selected as a model lactobacilli organism for further investigation. When the *L. brevis* was incubated with the crystalline cellulose Avicel, a significant portion of the bacterium adhered to the cellulose. The adsorption rate for the *L. brevis* grown in MRS/glucose (27.5%) was comparable to those of the bacteria grown in the MRS/WAX (27.8%) and the MRS/WAX+Xyn (20.8%). In the scanning electron microscopic (SEM) analysis, the *L. brevis* cells were clearly shown to be directly attached to the crystalline cellulose (Figure 2). These results indicated that *L. brevis* can, like other lactobacilli, adhere to PCWP. However, the mechanisms underlying the adhesion of lactobacilli to PCWP are poorly understood.

### 2.2. Four WxL Proteins and One S-Layer Protein Could Bind Either Cellulose or Xylan

A transcriptomic analysis of *L. brevis* indicated that the transcript abundance of 597 and 441 genes increased (>2-fold) in the MRS/WAX and MRS/WAX+Xyn, respectively, when compared with those in the MRS/glucose. The most up-regulated genes included those encoding xylosidases and arabinofuranosidases (data not shown), supporting the notion that *L. brevis* uses these enzymes to degrade xylooligosaccharides into fermentable sugars. In addition to these are the hypothetical proteins, WxL proteins, and S-layer proteins, whose functions have never been elucidated. It was also noted that the transcript levels of some of the WxL proteins, S-layer proteins, and hypothetical proteins were elevated to up to 14.6-fold in the MRS/WAX+Xyn medium when compared with those in the MRS/glucose (Table 1). This suggested that these proteins might play a role in *L. brevis*’ confrontation with the polysaccharide WAX. Eight out of the twelve proteins with unknown functions had an alkaline pI (Table 1).

**Figure 3:**
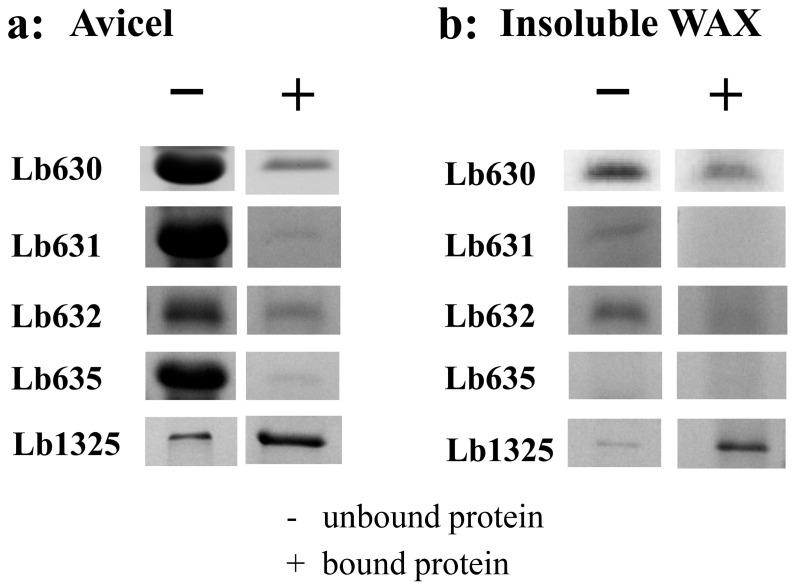
Binding of Lb630, Lb631, Lb632, Lb635, and Lb1325 to crystalline cellulose (**a**) and insoluble WAX (**b**).

Among these proteins, Lb630, Lb631, Lb632, Lb635, and Lb636 were annotated as WxL proteins, Lb634 was predicted to be an LPxTG protein, Lb1325 and Lb1145 were putative S-layer proteins, and the others were hypothetical proteins. Interestingly, all these proteins were also predicted to have a signal peptide, while the WxL proteins only contained a single domain. The WxL, LPxTG, S-layer, and hypothetical proteins were, therefore, predicted to be secreted and located on the cell surface, suggesting that they would have the chance to interact with PCWP. Therefore, the ability of the proteins to bind PCWP was investigated. The genes were individually cloned into pET-28a(+) and transformed in *E. coli* for recombinant expression. Among the proteins, only Lb630, Lb631, Lb632, Lb635, and Lb1325 were expressed successfully in a soluble form. The purified proteins were discovered to be able to bind the crystalline cellulose or insoluble wheat arabinoxylan (except Lb631 and Lb635 for WAX) albeit with apparently varying affinities (Figure 3). The seemingly universal binding ability of WxL proteins to plant cell wall polysaccharides has not been reported previously and is, therefore, intriguing, especially since they were from the same gene cluster (Supplementary Figure S1). Since, unlike the other WxL proteins, Lb630 was expressed and appeared to bind cellulose at a relatively high level, it was chosen for further study. 

### 2.3. Mutational Study of Lb630

In glycoside hydrolases and carbohydrate-binding modules (CBMs), aromatic residues are important residues that provide hydrophobic interactions with cellulose and xylan [22,23]. Lb630 bears three tryptophans, three phenylalanines, and two tyrosines. The multiple amino acid sequence alignment of Lb630 with b631, Lb632, Lb635, and other selected homologous proteins indicated that many of the aromatic residues were conserved (Supplementary Figure S2). The binding of crystalline cellulose is easier to monitor than that of insoluble WAX. Therefore, to understand whether any of the aromatic residues would participate in the binding to PCWP, a site-directed mutagenesis of each of these residues to alanine was carried out, and the mutants were tested for their binding ability to cellulose. While the mutants F30A, W61A, and W156A were the most impaired in binding, the others were moderately to slightly affected (Table 2). This suggested that F30, W61, and W156 were the most likely to be involved in the binding to crystalline cellulose. 

### 2.4. The Lb630 Homologs in E. faecalis Could Bind Cellulose and Xylan

**Figure 4:**
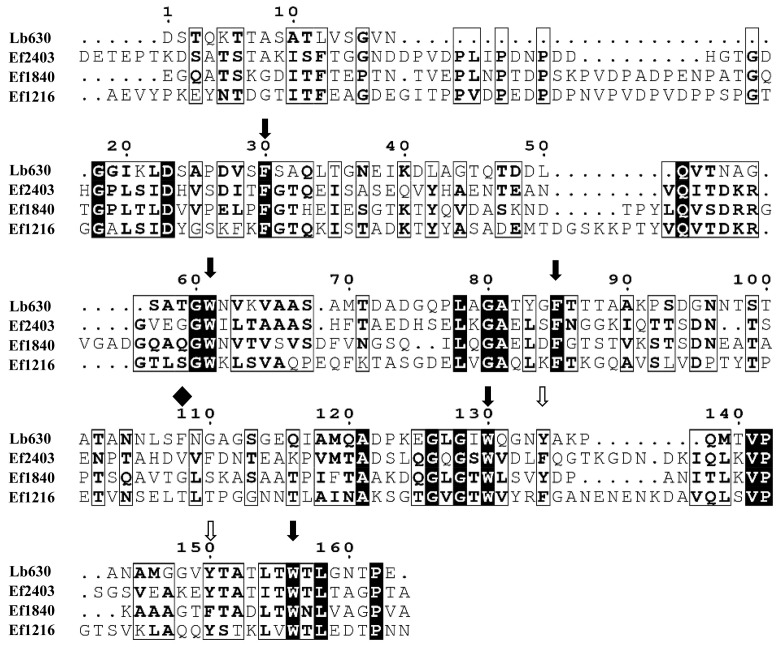
Amino-acid sequence alignment of Lb630 and its homologs in *E. faecalis*. Solid arrows represent strictly conserved aromatic amino acids, empty arrows are similar residues, and diamonds indicate non-conserved residues. Rectangle boxes with shadow indicate strictly conserved amino acids. Rectangular boxes are similar residues. The amino acids in bold letters represent identical and similar ones.

**Figure 5:**
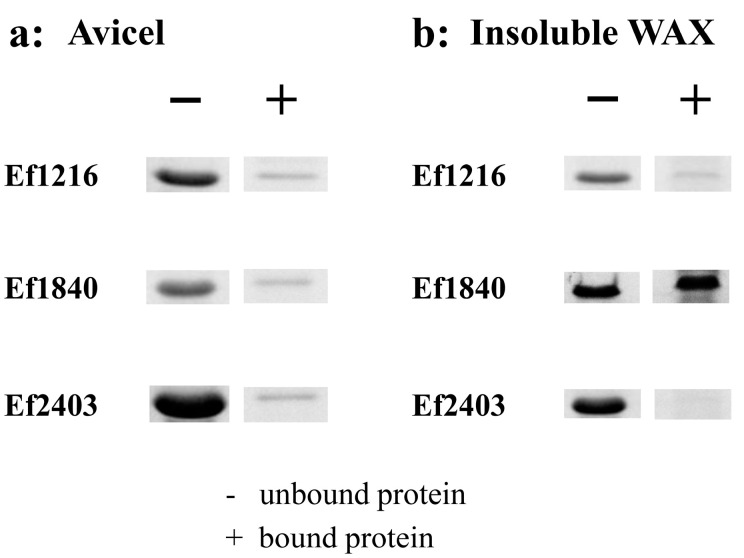
Binding of the Lb630 homologs in *E. faecalis* to cellulose and xylan. Binding of Ef1216, Ef1840, and Ef2403 to Avicel crystalline cellulose (**a**) and insoluble wheat arabinoxylan (**b**).

Because of the proposed contribution of the aromatic residues to binding, we hypothesized that WxL proteins from other gut bacteria with these conserved residues (particularly F30, W61, and W156) might also be able to bind cellulose and xylan. Three WxL proteins (Ef1216, Ef1840, and Ef2403) with low amino-acid sequence identity (28% to 30%) to the Lb630 but bearing identical counterparts of the three aromatic residues (F30, W61, and W156) were identified in the *E. faecalis* (Figure 4). These WxL proteins were also recombinantly expressed in the *E. coli*. As with the Lb630, all these proteins could bind to the crystalline cellulose and insoluble WAX (except Ef2403 with WAX, Figure 5).

## 3. Discussion

In this study, *L. brevis* was discovered to bind cellulose, which was similar to other gut-resident lactobacilli. The binding of xylan was not found under the conditions used in this study. Cellulose and xylan are well known to be intertwined in plant cell walls, and there are many PCWP-degrading glycoside hydrolases and lyases that do not degrade cellulose but bear a cellulose-binding domain [24]. The proximity of cellulose and xylan enables these enzymes to degrade other polysaccharides. Similarly, the binding of cellulose may also enable the bacterium *L. brevis* to make close contact with PCWP and, in particular, xylan.

Previous studies from other researchers have shown that the WxL domain may only act as an anchoring module, mediating non-covalent binding appendix proteins to the bacterial cell surface [25,26]. To the best of the authors’ knowledge, this is the first time that putative cell-surface WxL proteins from lactobacilli have been reported to bind PCWP. Note that the WxL domain-containing protein Lb630 appears to bind insoluble polysaccharides only (Supplementary Figure S3). This is similar to the type A surface-binding CBMs (discovered in families 1, 2a, 3, 5, and 10), which have a binding preference for insoluble, highly crystalline cellulose but show little or no affinity with soluble carbohydrates [23]. In these CBMs, aromatic residue mediated hydrophobic interaction plays a key role in the interactions of proteins with polysaccharides. Currently, there is no three-dimensional structure available for homologs of Lb630 (and other WxL proteins in *L. brevis*) with amino-acid sequence identities above 30%, preventing the homologous modeling of a structure for Lb630. However, AlphaFold2, RoseTTAFold, and other software provide a means for the de novo modeling of the structure [27,28]. With these two kinds of software, the Lb630 was predicted to form a compact structure with eight anti-parallel β-strands (Supplementary Figure S4). Two (F30 and W156) of the three aromatic residues that were likely to have played an important role in the binding were located on the surface (Supplementary Figure S4), and their side-chain groups appeared to form a flat platform that could make hydrophobic interactions with cellulose. However, without the crystal structure, the possibility of the occurrence of other interactions, such as the hydrogen bonding of the PCWP substrates with the polar residues in the Lb630, could not be excluded.

Although some surface-layer proteins were previously discovered to be able to bind PCWP, normally, this binding relied on carbohydrate-binding modules [29,30]. In addition, homologous proteins of Lb1325 (the surface-layer protein investigated in this study) in the non-redundant GenBank protein database have not been reported to bind PCWP, which is indicative of the novelty of the finding for the Lb1325 in our study. However, we noted that the binding of the WxL domain-containing proteins and the S-layer protein to the PCWP appeared not to be as strong as that of ordinary cellulose- and xylan-binding CBMs [31,32] as a large portion remained unbound under the incubation conditions we used. It remains to be elucidated if this was due to the sensitivity of the proteins to oxygen or components in the buffer, as observed in other *E. faecalis* WxL proteins [19]. Moreover, it was observed that the WxL proteins in *E. faecalis* can interact with each other in binding to human extracellular matrix proteins. Therefore, it is not impossible that some of the WxL proteins might assemble into complexes, which could increase the affinity of the WxL proteins. However, this requires further in vitro biochemical characterization, when the recombinant proteins can be prepared. Mutants that have Lb630 and even the entire gene cluster knocked out may provide more clues as to how these proteins would work together and affect the adhesion ability of the bacterium. In addition, the PCWP binding ability of different types of protein, including the WxL and S-layer proteins, strongly suggested that other cell surface proteins could also be involved. Those that were not successfully expressed and investigated in this study could produce stronger interactions with PCWP.

**Figure 6:**
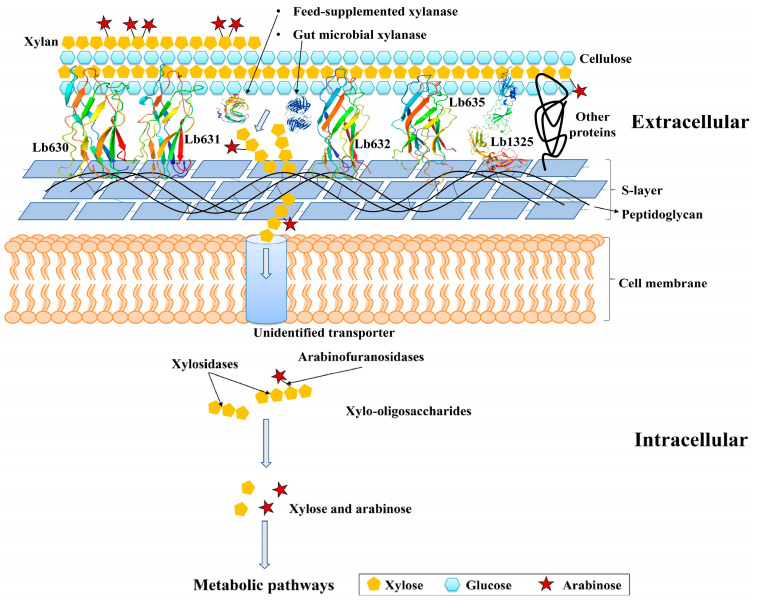
A model depicting how the WxL and S-layer proteins may help *L. brevis* to utilize xylooligosaccharides. For simplification, wheat arabinoxylan was shown to represent dietary xylan. The WxL and S-layer proteins were presented as modeled structures using AlphaFold2 or RoseTTAFold.

The adhesion of cells to PCWP may enable the rapid assimilation of degraded saccharides into the interior of bacteria, thereby preventing nutrient loss and assisting in the establishment of growth competence in a highly competent environment. The adhesion to PCWP has been observed in gut bacteria, such as *F. succinogenes* and *R. albus* [33], that can utilize plant cell wall polysaccharides. Importantly, it has been reported that cell surface protein mediated adhesion is involved in the ‘selfish’ utilization of mannan by *Bacteroides thetaiotaomicron*, thereby favoring its growth in the gut niche. Lactobacilli are xylooligosaccharide-utilization bacteria, but their adhesion to PCWP is similarly well documented [15,16,34,35]. Therefore, based on the knowledge gained through the current study and by analogy to the PCWP-utilizing gut bacteria, a preliminary model could be proposed to describe how *L. brevis* might similarly utilize the xylo-oligosaccharides derived from PCWP with the assistance of WxL, S-layer proteins, and, as is likely, other cell-surface proteins as well. As shown in Figure 6, *L. brevis* might adhere to cellulose in PCWP via WxL proteins, surface-layer proteins such as Lb1325, and other, unidentified, proteins. The xylanases encoded by gut microbes or used as feed additives degrade xylan into xylo-oligosaccharides. The adhesion of bacterial cells to PCWP favors the capturing of the released xylo-oligosaccharides by the bacterium, which is supposed to facilitate their transportation across the cell membranes of *L. brevis* through unidentified sugar transporters. In the cytoplasm, the xylosidases, arabinofuranosidases, α-glucuronidases, and esterase cooperate to degrade xylooligosaccharides into fermentable sugars, such as xylose and arabinose, for entry into the metabolic pathways. However, since this was beyond the scope of the current study, further investigations are needed in the future to verify the proposed model. The experiments may include the biochemical characterization of glycoside hydrolases for their catalytic activities against heterogenous xylo-oligosaccharides, the verification of the localization of the putative cell surface proteins, the determination of the putative cooperation among the cell surface proteins, the measurement of the adhesion ability of the *L. brevis* mutants with deletions of one or multiple genes encoding the surface proteins, and a comparison of the mutant and wild-type strains for their ability to outgrow other gut bacteria in co-cultures. The data collected from all these experiments will present more insights and solidify the role of cell surface proteins in helping *L. brevis* to acquire carbon sources and energy in the highly competitive gut niche.

## 4. Materials and Methods

### 4.1. Microbial Strains and Culture

The *Escherichia coli* Trans 1-T1 strain (Transgen, Beijing, China) was used for plasmid construction and propagation in this study. The *E. coli* BL21(DE3) (Transgen, Beijing, China) was used for recombinant expression of selected genes. All *E. coli* strains were grown in a Luria–Bertani (LB) medium. The *L. brevis* and *E. faecium* were isolated from the intestinal content of broiler chickens and cultured in the De Man, Rogosa, and Sharpe (MRS) medium under anaerobic condition at 37 °C.

### 4.2. Binding of L. brevis to Cellulose and Xylan

The adhesion to insoluble PCWP was measured by co-sedimentation assay according to the method described in [36]. *L. brevis* was cultured in the MRS medium for 2 d. After centrifugation, cells were transferred to fresh MRS media containing different carbon sources, including glucose, wheat arabinoxylan (WAX), and WAX plus xylanase. The culture was continued for 6 h. The cells were then washed twice with a PC buffer (10 mmol/L citric acid-Na_2_HPO_4_, pH 5.0, 75 mmol/L NaCl) and suspended in the same buffer at an OD_600_ of 0.7. An equal volume (0.5 mL each) of cell suspensions and 50 mg Avicel (Sigma-Aldrich, St. Louis, MI, USA) or insoluble WAX (Megazyme International, Bray, Ireland) suspensions were mixed. The mixtures were shaken end-over-end slowly for 30 min at room temperature. The bacteria-cellulose suspension was placed at room temperature for 30 min, and then 200-microliter samples were taken from 0.3 cm below the liquid level for the determination of the absorbance at 600 nm. The adhesion rate of the bacteria to cellulose and xylan was calculated as follows: [100% × (1 − (a − b)/c)], where “a” represents the OD_600_ value of the supernatant of bacteria incubated with a polysaccharide, “b” represents the value of the control containing only a polysaccharide but no bacteria, and “c” represents the value of the control containing bacteria but no polysaccharide.

### 4.3. Scanning Electron Microscopy

*L. brevis* cells and Avicel crystalline cellulose suspension, both diluted at an appropriate ratio, were slowly filtered to 0.45 μm filter membrane, and slowly washed with sterile 0.9% sodium chloride solution. The filter membranes were removed for vacuum freeze-drying overnight after freezing at −80 °C for half an hour, and secured onto the aluminum stubs of the scanning electron microscope with sticky tabs. Next, the samples were coated with gold in an EM ACE600 sputter coater (Leica, Vienna, Austria) and viewed with the emission scanning electron microscope SU3500 (Hitachi High-Tech Co., Tokyo, Japan). The micrographs were taken at a constant voltage of 5.0 kV.

### 4.4. Growth of L. brevis in MRS Media Supplemented with Different Carbon Sources

A single colony of *L. brevis* was inoculated into the MRS liquid medium and the culture was carried out under anaerobic conditions at 37 °C for 2 d. After centrifugation, the bacteria were washed with sterile 0.9% sodium chloride solution three times, and the pellets were re-suspended in sterile water. The OD_600_ of the cell suspension was adjusted to 1.5, and the cells (3% *v*/*v*) were inoculated to liquid MRS containing glucose, WAX, or WAX supplemented with a commercially available xylanase (Hexin, Jining, China). The OD_600_ was determined by using a GENESYS 30 spectrophotometer (Thermo Fisher Scientific, Waltham, MA, USA).

### 4.5. Genome Sequencing

*L. brevis* and *E. faecium* were cultured in liquid MRS medium under anaerobic conditions at 37 °C for 2 d. Next, the bacteria were collected and the genomic DNAs were extracted by using a FastPure Bacteria DNA Isolation Mini Kit (Vazyme, Nanjing, China). The genomic DNAs were used for genome sequencing (Allwegene, Beijing, China). The DNA purity and integrity were analyzed by agarose gel electrophoresis and quantified by Qubit 2.0 fluorometer. Subsequently, sequencing libraries were constructed using the NEBNext Ultra DNA Library Prep Kit (New England Biolabs, Ipswich, MA, USA), and were further sequenced using Illumina HiSeq/NovaSeq PE150 platform. Finally, all good-quality reads were assembled into longer scaffolds using SPAdes (v3.13.0) software, and scaffolds larger than 500 bp were selected for subsequent functional analysis.

### 4.6. Transcriptome Sequencing

*L. brevis* was grown in the MRS media containing glucose, WAX, or WAX supplemented with the xylanase, respectively. The culture was continued for 15 h. Next, the bacteria were pelleted and the total RNAs under the three conditions were extracted from the collected bacteria using the TRIZOL reagent (Invitrogen, Waltham, MA, USA), according to the manufacturer’s instructions. The total RNAs were used for RNAseq analysis (Allwegene, Beijing, China). Briefly, RNA was quantified by using a NanoDrop 2000 spectrophotometer (Implen, CA, USA) and checked for its quality using an Agilent 2100 Bioanalyzer (Agilent Technologies, Santa Clara, CA, USA). The mRNA was enriched by removing rRNAs using a Ribo-off rRNA Depletion Kit (Bacteria) (Vazyme, Nanjing, China). After enrichment, the sequencing library was constructed using the NEBNext Ultra RNA Library Prep Kit (New England Biolabs, Ipswich, MA, USA). Double-stranded cDNAs were synthesized by reverse transcription using random primers, followed by synthesis of the second cDNA strand. The products were purified, terminally repaired, and added with a single adenosine. The cDNA library was finally created by PCR amplification and sequenced on an Illumina Hiseq 4000 platform, in which 150-bp paired-end reads were generated with 2 Gb clean data.

### 4.7. Structural Prediction of the WxL and S-Layer Proteins

The three-dimensional structures of Lb630, Lb631, Lb632, Lb635, and Lb1325 were predicted using AlphaFold2 [27] and RoseTTAFold [28].

### 4.8. Cloning, Expression, and Purification of Recombinant Proteins

The *Lb64, Lb630, Lb631, Lb632, Lb634, Lb635, Lb636,* *Lb1145, Lb1325, Lb1800, Lb2458, Lb2498, Ef1216, Ef1840*, and *Ef2403* genes were amplified from the genomic DNAs of *L. brevis* and *E. faecium*, respectively, by polymerase chain reaction (PCR) using gene-specific primer pairs (Supplementary Table S1). The amplified DNAs were inserted into the *Nde*I and *Not*I restriction sites of the pET-28a(+) plasmid to obtain pET28a-Lb64, pET28a-Lb630, pET28a-Lb631, pET28a-Lb632, pET28a-Lb634, pET28a-Lb635, pET28a-Lb636, pET28a-Lb1145, pET28a-Lb1325, pET28a-Lb2458, pET28a-Lb2498, pET28a-Ef1216, pET28a-Ef1840, and pET28a-Ef2403, respectively. The recombinant plasmids were transformed into *E. coli* BL21(DE3) competent cells. The positive transformants were cultured in LB overnight at 37 °C. The pre-culture was transferred to 300 mL fresh LB medium and the culture was continued for 3 h. When the OD_600_ reached 0.6–0.8, a final concentration of 0.5 mmol/L of isopropyl-β-D-thiogalactopyranoside (IPTG) was added to the cells for inducing recombinant proteins, and the culture was carried out at 16 °C for 15 h. The recombinant proteins were purified by immobilized metal ion affinity chromatography. The soluble extract was passed through a Ni-affinity column resin and the resin was washed with a binding buffer (20 mmol/L Tris-HCl, pH 7.4, 500 mmol/L NaCl). The bound proteins were eluted using the elution buffer (20 mmol/L Tris-HCl, pH 7.4, 500 mmol/L NaCl, and 500 mmol/L imidazole). The fractions with pure proteins were pooled, concentrated, and changed to a protein-storage buffer (50 mmol/L Tris-HCl, pH 7.4, 150 mmol/L NaCl).

### 4.9. Binding of the Proteins to Insoluble Polysaccharides

To measure the ability of the proteins to bind insoluble polysaccharides, 100 μg of recombinant WxL proteins (Lb630, Lb631, Lb632, and Lb635), the S-layer protein Lb1325, the wild-type Lb630, its mutants, and the *E. faecium* proteins were individually mixed with 30 mg of insoluble Avicel or WAX in the protein-storage buffer. The mixtures were shaken end-over-end for 1 h at 4 °C. Next, the insoluble polysaccharides were precipitated by centrifugation. The precipitate was washed with 1 mL protein storage buffer four times, re-suspended with 70 μL of SDS-PAGE loading buffer, and then boiled for 5 min to release the bound protein. One tenth the volume of the supernatant (unbound protein) and precipitate (bound protein) were analyzed by SDS-PAGE electrophoresis. The gels were run in the Criterion™ Vertical Electrophoresis Cell (Bio-Rad, Hercules, CA, USA) at 120 V for 80 min. The gels were stained in a solution of Coomassie Bright Blue G-250 for 20 min and destained in a solution containing 10% ethanol and 10% acetic acid 2–3 times. Subsequently, the decolorized gels were visualized and analyzed using the ChemiDoc™ XRS+ system (Bio-Rad, Hercules, CA, USA). Next, the amount of target protein was quantified by ImageJ software. 

### 4.10. Affinity Gel Electrophoresis

Affinity gel electrophoresis was used to determine whether Lb630 could bind to soluble polysaccharides. The gel was prepared according to the method described previously [37]. Briefly, the upper layer of gel was composed of 3% acrylamide in 1.5 mol/L pH 8.3 Tris-HCl buffer. The lower layer was composed of 12% acrylamide in the same buffer, supplemented with 0.15 mg/mL of WAX, sodium carboxymethyl cellulose (CMC-Na) (Sigma-Aldrich, St. Louis, MI, USA), pectin (Sigma, St. Louis, MI, USA), and other soluble polysaccharides. The buffer consisted of 25 mM Tris-HCl (pH 8.3) and 250 mM glycine, and the electrophoresis was carried out at 4 °C for 4 h. The proteins were visualized by Coomassie Bright Blue G-250 staining.

### 4.11. Site-Directed Mutagenesis

Site-directed mutagenesis of Lb630 was carried out using a Mut Express II Fast Mutagenesis Kit (Vazyme, Nanjing, China) by following the instructions of the manufacturer. The pET28a-Lb630 plasmid was used as the template. After PCR amplification, the *Dpn*I restriction enzyme was added to digest the template plasmid. Then the reaction products were used to transform the *E. coli* Trans1-T1 competent cells. The plasmids bearing the mutations were sequenced for their integrity. The sequences of the primers for mutagenesis are listed in Supplementary Table S1.

## 5. Conclusions

The genes encoding WxL proteins and S-layer proteins were highly transcribed in a *L. brevis* strain cultured in an MRS medium supplemented with wheat arabinoxylan and xylanase. The recombinant proteins of Lb630, Lb631, Lb632, Lb635, and Lb1325 were able to bind either cellulose or xylan. A mutational analysis suggested the importance of three aromatic residues in the WxL protein Lb630, based on which three homologous WxL proteins from *E. faecalis* bearing the identical counterparts of the three aromatic residues were further identified to similarly bind the cellulose and xylan. The WxL proteins and surface-layer proteins were able to contribute to the adhesion of *L. brevis* to PCWP, which probably facilitated the assimilation of the xylo-oligosaccharides by this gut bacterium.

## Figures and Tables

**Table 1:** Twelve most up-regulated genes with unknown functions from *L. brevis* cultured in MRS/WAX+Xyn ^a^.

No.	ORF ID	Accession No. of Closest Homologs in GenBank	Transcript Abundance (FPKM ^b^)			Annotation	pI	Signal Peptide	Domain Structure 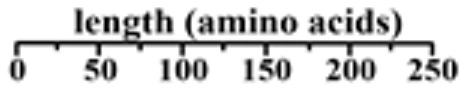
			WAX+Xyn (WE)	Glucose (G)	WE/G				
1	Lb1325	WP_084995011.1	62,059	68,801	<1	S-layer protein	9.51	Yes	
2	Lb1800	WP_015473174.1	10,592	727	14.6	hypothetical protein	10.37	Yes	
3	Lb1145	WP_087716548.1	9932	5603	1.6	S-layer protein	9.62	Yes	
4	Lb631	WP_087717374.1	9074	2695	3.4	WxL protein	4.64	Yes	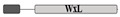
5	Lb636	WP_085763449.1	6907	2262	3.1	WxL protein	9.55	Yes	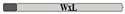
6	Lb635	WP_085763450.1	6499	1788	3.6	WxL protein	4.41	Yes	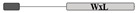
7	Lb2498	WP_035464648.1	6299	3522	1.8	hypothetical protein	10.4	Yes	
8	Lb632	WP_087717372.1	5741	1161	4.9	WxL protein	4.7	Yes	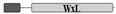
9	Lb634	WP_085763451.1	3796	1015	3.7	LPxTG protein	9.85	Yes	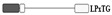
10	Lb64	WP_043022000.1	2497	1597	1.6	peptidoglycan lytic protein P45	9.77	Yes	
11	Lb2458	AJA80418.1	2412	1902	1.3	hypothetical protein	9.81	Yes	
12	Lb630	WP_085763455.1	1856	673	2.8	WxL protein	4.58	Yes	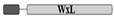

^a^ The black square indicates the predicted signal peptide. ^b^ FPKM: fragments per kilobase of exon model per million mapped fragments.

**Table 2:** Relative affinities of Lb630 and its mutants to cellulose.

Protein	Bound/Unbound (×100) ^a^	Relative Affinity (%) ^b^
Wild-type	26.3 ± 2.0	100
F30A	13.7 ± 0.5	52
W61A	11.4 ± 1.2	43
F85A	22.8 ± 4.0	86
F108A	17.2 ± 0.9	65
W130A	15.1 ± 0.6	57
Y134A	15.4 ± 1.4	59
Y150A	19.1 ± 1.3	72
W156A	12.4 ± 0.5	47

^a^ Bound/unbound (%) was calculated by dividing the intensity of the band in the bound fraction by that in the unbound fraction for wild-type Lb630 and its mutants. Values are reported as means ± standard deviations. ^b^ Relative affinity was calculated by dividing the values of bound/unbound of the mutant by that of the wild type.

## Data Availability

All data supporting the conclusions of this article are included within the manuscript.

## Supplementary Materials

The following supporting information can be downloaded at: https://www.mdpi.com/article/10.3390/ijms23084136/s1.
