# Supplementary material for: Identification of WxL and S-Layer Proteins from Lactobacillus brevis with the Ability to Bind Cellulose and Xylan
Source: Int J Mol Sci. 2022 Apr 8;23(8):4136. doi: 10.3390/ijms23084136 (PMC9026416; doi:10.3390/ijms23084136)
Supplement: Supplementary file 1 [file ijms-23-04136-s001.zip › ijms-1634628-supplementary.pdf]

International Journal of
Molecular Sciences

MDPI

Supplementary materials

Identification of WxL and S-layer proteins from *Lactobacillus brevis* with the ability to bind cellulose and xylan

Zhenzhen Hao, Wenjing Zhang, Xiaolu Wang, Yuan Wang, Xing Qin, Huiying Luo, Huoqing Huang*, Xiaoyun Su*

State Key Laboratory of Animal Nutrition, Institute of Animal Sciences, Chinese Academy of Agricultural Sciences, Beijing 100193, China; zhenzhenhao2012@163.com (Z.H.); 15927146115@163.com (W.Z.); xiaolu4444@126.com (X.W.); wangyuan08@caas.cn (Y.W.); qinxing@caas.cn (X.Q.); luohuiying@caas.cn (H.L.)
* Correspondence: huanghuoqing@caas.cn (H.H.); suxiaoyun@caas.cn (X.S.)

Supplementary Table S1. Primers used in this study.

Primer	Sequence (5′–3′)	Usage
PLb630F	tggtgccgcgcggcagcGACTCAACCCAAAAGACCAC	Cloning of *Lb630* into pET-28a(+)
PLb630R	ggtggtggtggtgctcgagtTTATTCCGGCGTATTACCCAACGTCCACG	Cloning of *Lb630* into pET-28a(+)
PLb631F	ctggtgccgcgcggcagcGCTGATTTTGGGACAACT	Cloning of *Lb631* into pET-28a(+)
PLb631R	tggtggtggtgctcgagtTTATGCCTTTACGGAATCAACCAA-GCTCCAG	Cloning of *Lb631* into pET-28a(+)
PLb632F	gcctggtgccgcgcggcagcGCTAATACAACTTCGGC	Cloning of *Lb632* into pET-28a(+)
PLb632R	ggtggtggtgctcgagtTTAGTTTACTGAGTTAGCCAAC-GTCCAAGTTA	Cloning of *Lb632* into pET-28a(+)
PLb634F	cggcctggtgccgcgcggcagcGATGTTCAGCAATCTACTTA	Cloning of *Lb634* into pET-28a(+)
PLb634R	ggtggtggtgctcgagtTTAATTTTCTTGCTTCCTACGTTTTAACAACA	Cloning of *Lb634* into pET-28a(+)
PLb635F	cctggtgccgcgcggcagcGCTACTAGTGACAGTGCT	Cloning of *Lb635* into pET-28a(+)
PLb635R	ggtggtggtggtgctcgagtTTAGAATGGGGCGTTCTTTAGAG-TCCAAG	Cloning of *Lb635* into pET-28a(+)
PLb1325F	gcctggtgccgcgcggcagcAAGAGCTATGCAACTG	Cloning of *Lb1325* into pET-28a(+)
PLb1325R	ggtggtggtgctcgagtTTAAGCAATCCAGCTGTTATCTGGCTTACTG T	Cloning of *Lb1325* into pET-28a(+)
F30A-F	TCCGCCCCAGATGTTAGTGCGTCCGCGCAGT	Creation of the F30A mutant of Lb630
F30A-R	CGCACTAACATCTGGGGCGGAGTCTAACTTGATCCCACC	Creation of the F30A mutant of

		Lb630
W61A-F	GCCGGCTCGGCAACTGGTGCGAACGTAAAGG	Creation of the W61A mutant of Lb630
W61A-R	CGCACCAGTTGCCGAGCCGGCATTGGTTAC	Creation of the W61A mutant of Lb630
F85A-F	TATGGGGCAACAACTACAGCGGCTAAACCCTC	Creation of the F85A mutant of Lb630
F85A-R	AGTTGTTGCCCCATATGTTGCACCAGCTAACG	Creation of the F85A mutant of Lb630
F108A-F	CGACTGCTAACAATCTCAGCGCGAACGGTGCTGGTAGT	Creation of the F108A mutant of Lb630
F108A-R	CGCGCTGAGATTGTTAGCAGTCGCCGTAGAGGTGTTATTTCC	Creation of the F108A mutant of Lb630
W130A-F	GATCCCAAAGAAGGCTTAGGCATTGCGCAAGGTAACTATGC	Creation of the W130A mutant of Lb630
W130A-R	CGCAATGCCTAAGCCTTCTTTGGGATCAGCTTGCAT	Creation of the W130A mutant of Lb630
Y134A-F	GCTTAGGCATTTGGCAAGGTAACGCGGCCAAACCACAA	Creation of the Y134A mutant of Lb630
Y134A-R	CGCGTTACCTTGCCAAATGCCTAAGCCTTCTTTGGGATCAG	Creation of the Y134A mutant of Lb630
Y150A-F	CGCCATGGGTGGTGTGGCGACGGCAACAT	Creation of the Y150A mutant of Lb630
Y150A-R	CGCCACACCACCCATGGCGTTAGCTGGCAC	Creation of the Y150A mutant of Lb630
W156A-F	TACACGGCAACATTGACGGCGACGTTGGGTAAT	Creation of the W156A mutant of Lb630
W156A-R	CGCCGTCAATGTTGCCGTGTACACACCACCCAT	Creation of the W156A mutant of Lb630
PEf2403F	gtgccgcgcggcagcGATGAAACAGAGCCAACCAAAG	Cloning of *Ef2403* into pET-28a(+)
PEf2403R	tggtggtgctcgagtCTAAGCAGTTGGGCCTGCTGTGA	Cloning of *Ef2403* into pET-28a(+)
PEf1840F	gtgccgcgcggcagcGAAGGTCAAGCAACTTCAAAAGG	Cloning of *Ef1840* into pET-28a(+)
PEf1840R	tggtggtgctcgagtCTATGCTACTGGGCCAGCCACGAGA	Cloning of *Ef1840* into pET-28a(+)
PEf1216F	gtgccgcgcggcagcGCTGAAGTATACCCAAAGGAA	Cloning of *Ef1216* into pET-28a(+)
PEf1216R	ggtggtggtggtgctcgagtCTAGTTATTAGGTGTATCTTCTAATGTCC	Cloning of *Ef1216* into pET-28a(+)

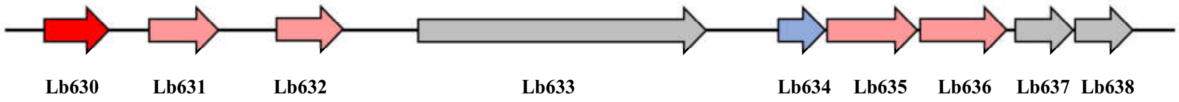

Supplementary Figure S1. The gene cluster encoding the WxL proteins (Lb630, Lb631, Lb632, Lb635) investigated in this study.

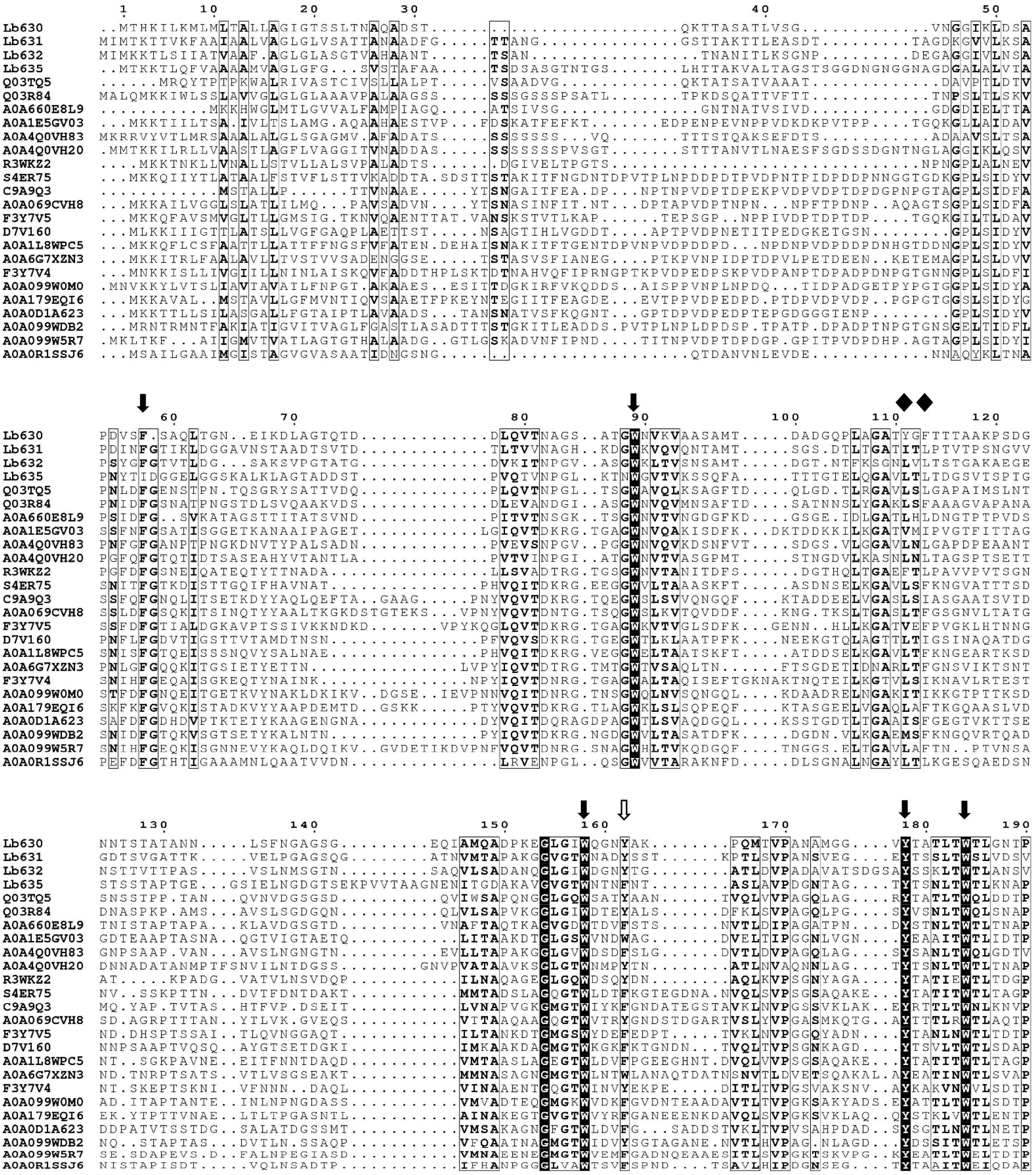

Supplementary Figure S2. Multiple amino acid sequence alignment of Lb630 with homologous proteins in other microbes. Solid arrows represent conserved aromatic amino acids, empty arrows are similar residues, and diamonds indicate non-conserved resi-dues. The origins of the proteins are: Lb630, *L. brevis*; Lb631, *L. brevis*; Lb632, *L. brevis*; Lb635, *L. brevis*; Q03TQ5: *Levilactobacillus brevis;* Q03R84: *Levilactobacillus brevis;* A0A660E8L9: *Lactiplantibacillus mudanjiangensis;* A0A1E5GV03: *Enterococcus quebecensis;* A0A4Q0VH83: *Levilactobacillus suantsaii;* A0A4Q0VH20: *Levilactobacillus suantsaii;* R3WKZ2: *Enterococcus phoeniculicola* ATCC BAA-412; S4ER75: *Enterococcus faecalis* 13-SD-W-01; C9A9Q3: *Enterococcus casseliflavus* EC20; A0A069CVH8: *Weissella oryzae*; F3Y7V5: *Melissococcus plutonius*; D7V160: *Listeria grayi* DSM 20601; A0A1L8WPC5: *Enterococcus ratti*; A0A6G7XZN3: *Erysipelothrix* sp. HDW6C; F3Y7V4: *Melissococcus plutonius*; A0A099W0M0: *Listeria booriae*; A0A179EQI6: *Enterococcus thailandicus*; A0A0D1A623: *Paucilactobacillus wasatchensis*; A0A099WDB2: *Listeria booriae*; A0A099W5R7: *Listeria booriae*; A0A0R1SSJ6: *Lactobacillus versmoldensis* DSM 14857.

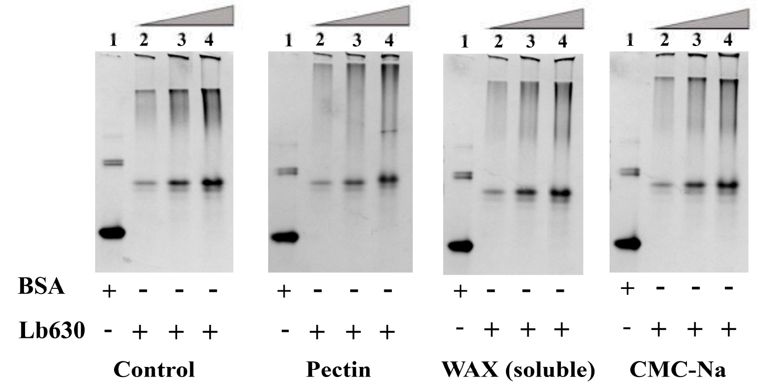

Supplementary Figure S3. Lb630 did not bind soluble polysaccharides including pectin, soluble wheat arabinoxylan, and sodium carboxymethyl cellulose (CMC). In lanes 2-4, 2, 4, and 8 μg Lb630 were added, respectively. Control: no polysaccharide was added to the gel. CMC-Na: sodium carboxymethyl cellulose; BSA: bovine serum albumin.

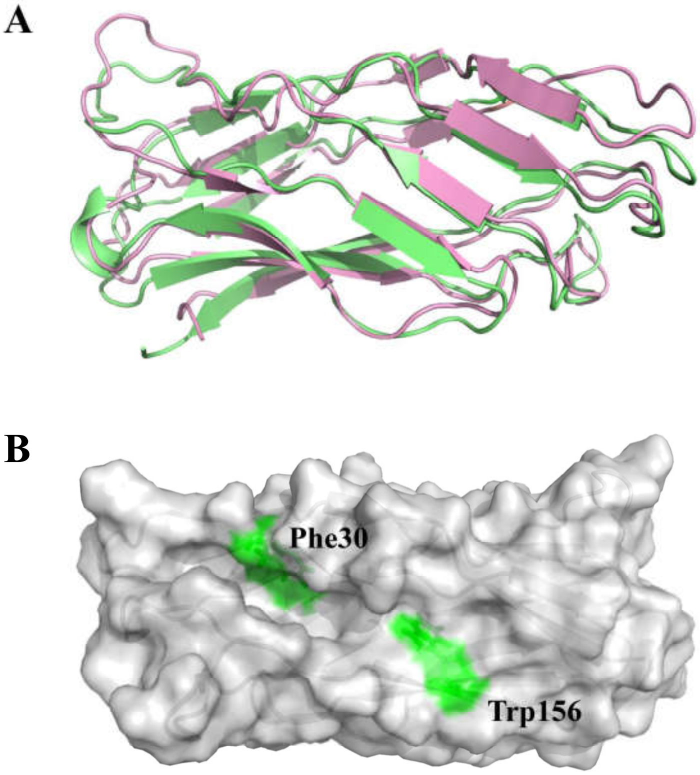

Supplementary Figure S4. Structural analysis of Lb630. A. Structural prediction of Lb630 using AlphaFold2 (pink) and RoseTTAFold (green). B. Two (F30 and W156) of the three aromatic residues that likely play most important roles in cellulose and xylan binding are present on the surface and labeled in green.
